# Recurrent Steroid‐Responsive Epiglottitis Following Cord Blood Transplantation: A Case Report

**DOI:** 10.1002/jha2.70441

**Published:** 2026-10-21

**Authors:** Ryo Nakagawa, Koichi Onodera, Hikaru Kon, Mizuki Watanabe, Hiroaki Abe, Eiji Ogawa, Hikaru Kumagai, Toaki Maeda, Akihisa Kawajiri, Shunsuke Hatta, Risako Kakuta, Noriko Fukuhara, Hideo Harigae

**Affiliations:** ^1^ Department of Hematology Tohoku University Hospital Sendai Miyagi Japan; ^2^ Department of Otolaryngology, Head and Neck Surgery Tohoku University Hospital Sendai Miyagi Japan

**Keywords:** ATL, cord blood transplantation, epiglottitis

## Abstract

Epiglottitis is an acute inflammation of the epiglottis, typically caused by bacterial infections and potentially life‐threatening due to rapid airway obstruction. There are extremely rare case reports of epiglottitis as a complication following allogeneic stem cell transplantation (allo‐SCT). We report a 56‐year‐old female case with adult T‐cell lymphoma (ATL) who suffered from recurrent epiglottitis after cord blood transplantation (CBT). Her epiglottitis was refractory to empirical antibiotic therapy. However, it responded promptly to corticosteroids, preventing the development of severe respiratory failure. This report implicated the importance of recognizing non‐infectious epiglottic inflammation in the setting of transplantation and cellular therapy.

**Trial Registration**: The authors have confirmed clinical trial registration is not needed for this submission.

## 1 Introduction

Epiglottitis is an inflammatory condition of the epiglottis, most commonly associated with bacterial infection, and can rapidly progress to severe airway compromise. In case with extremely severe swelling of the epiglottis, airway intervention including tracheostomy is often required [1]. Therefore, early diagnosis and prompt therapeutic intervention are crucial to improving clinical outcomes. To date, epiglottitis following allo‐SCT has rarely been reported, and its clinical features remain unclear. Here, we present a case of recurrent epiglottitis after CBT that was resistant to empirical anti‐bacterial/fungal therapy but showed a favorable response to treatment with corticosteroids.

## 2 Case Presentation

The case was a 56‐year‐old woman with newly diagnosed ATL involving the left cervical lymph nodes. She received six cycles of BV‐CHP (brentuximab vedotin, cyclophosphamide, doxorubicin and prednisolone), achieving complete remission. She subsequently underwent CBT from an HLA 4/6 matched donor. The conditioning regimen consisted of fludarabine (25 mg/m^2^ for 2 days), melphalan (40 mg/kg for 2 days), and total‐body irradiation (4 Gy in two fractions). Graft‐versus‐host disease (GVHD) prophylaxis included tacrolimus and short‐term methotrexate. Neutrophil engraftment was achieved by Day 20, and complete donor chimerism was confirmed on Day 28. On Day 17, the patient developed a localized erythematous rash, which was diagnosed as stage 1 cutaneous GVHD and treated with topical corticosteroids and concurrently presented symptoms of nausea. On Day 36, she presented with hoarseness, dysphagia, and dry cough, which rapidly worsened over several days. Flexible fiberoptic laryngoscopy showed marked swelling of the epiglottis and bilateral arytenoids, leading to a diagnosis of epiglottitis (Figure 1A,B). The airway was maintained, and conservative treatment was initiated with close monitoring and preparation for emergency tracheostomy. Computed tomography showed no abscess formation, deep neck infection, tumor recurrence, or other structural causes. At the onset of epiglottitis, the white blood count was 4 × 10^3^ /µL, with no left shift or elevation in CRP (0.21 mg/dL). Microbiological evaluation did not identify any bacterial, viral, or fungal pathogen, and blood cultures were negative. Because infectious epiglottitis remained a concern in the early post‐transplant period, empirical antimicrobial therapy was modified from cefepime and levofloxacin to meropenem and vancomycin, while micafungin was continued; however, her symptoms and laryngoscopic findings did not improve. Owing to progressive upper airway symptoms, dexamethasone (6.6 mg/day) was initiated for two days and then tapered, resulting in prompt symptomatic and laryngoscopic improvement (Figure 1C,D). However, on Day 57, she experienced recurrent dysphagia. Laryngoscopy confirmed relapsed epiglottitis (Figure 1E,F). No new infectious focus was identified. Given the previous lack of response to antimicrobial therapy and the prompt corticosteroid response, a similar corticosteroid regimen was reintroduced, again resulting in rapid improvement (Figure 1G,H). Subsequent low‐dose oral prednisolone maintenance (10 mg/day) successfully prevented further recurrence. On Day 130, she remained free of epiglottitis recurrence with maintenance therapy of prednisolone (7.5 mg/day) without evidence of ATL relapse.

**FIGURE 1:**
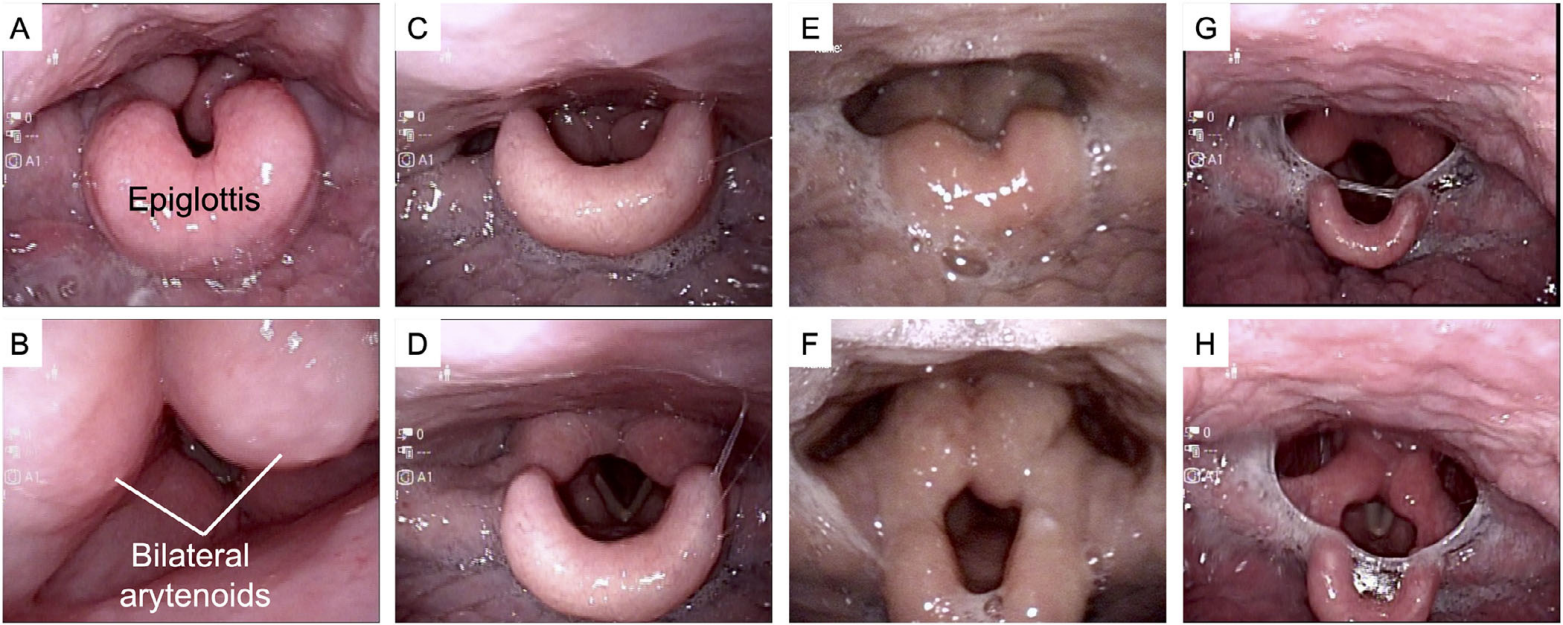
Images of flexible fiberoptic laryngoscopy. The upper images show swelling of the epiglottis, while the lower images depict swelling of bilateral arytenoids. (A, B) Laryngoscopic findings at the onset of epiglottitis on day 36 after transplantation. (C, D) Findings on day 48 after transplantation, showing temporary improvement of epiglottitis. (E, F) Findings on Day 57 after transplantation, indicating a relapse of epiglottitis. (G, H) Finding on Day 69 after transplantation, showing complete recovery of epiglottitis.

## 3 Discussion

Only a few cases of epiglottitis after allogeneic stem cell transplantation have been reported, including a case attributed to conditioning‐related mucosal toxicity [2], and the other to fungal infection [3]. In contrast, our patient developed symptoms nearly four weeks post‐transplant without preceding mucosal damage or identifiable infection. Recurrent epiglottitis in this patient occurred following cutaneous GVHD, and responded promptly to steroid therapy on each occasion. No identifiable infectious focus was found, and the epiglottitis was refractory to empirical antibacterial and antifungal therapy. Recurrence despite ongoing antimicrobial treatment, followed by recurrence after corticosteroid tapering and a reproducible response to corticosteroids, favors an inflammatory rather than an infectious mechanism. Post‐transplant lymphoproliferative disease (PTLD) was considered as a potential post‐transplant complication. However, neck CT scan showed no mass lesion, asymmetric thickening, or cervical lymphadenopathy in this case, unlike previous reported laryngeal PTLD cases [4, 5]. No subsequent laryngeal or lymph node lesions developed during follow‐up.

Steroids are administered for the treatment of epiglottitis in clinical practice [6], and symptoms in previously reported cases generally resolved within several days without recurrence [7, 8]. On the other hand, our case showed early recurrence following discontinuation of corticosteroids despite continued antibiotic treatment. One limitation of this report is that histological evaluation could not be performed due to the high risk of precipitating airway compromise; thus, a definitive diagnosis of GVHD or alloimmune epiglottitis could not be established, and infection cannot be completely excluded. Previous report described cases that developed localized oral acute GVHD following allogeneic HSCT, in which noninfectious, alloreactive lesions involved the oral mucosa, particularly the buccal mucosa and tongue [9]. Similar upper airway edema has been recently reported after CD19 or BCMA‐targeted chimeric antigen receptor (CAR) T‐cell therapy, where local cytokine‐mediated inflammation was suspected [10, 11, 12, 13]. In all cases, the cytokine‐related symptoms (CRS) resolved with steroid therapy in addition to IL‐6 receptor antagonist. Although the post‐transplant setting differs from CAR T‐cell therapy, this case may implicate a steroid‐responsive non‐infectious inflammatory mechanism following CBT, similar to local CRS. Epiglottitis has been reported in association with autoimmune disease [14]. Although specific diagnostic testing was not performed in this case, these conditions should be considered in similar cases, particularly when an infectious etiology is not identified.

## 4 Conclusion

In conclusion, we present a rare case of epiglottitis after CBT as a non‐infectious complication. Appropriate diagnostic evaluation by flexible fiberoptic laryngoscopy and early therapeutic intervention with corticosteroids prevented severe airway obstruction. Clinicians should recognize recurrent epiglottitis as a potentially life‐threatening complication after allo‐SCT. Careful airway assessment, exclusion of infection and structural causes, and timely consideration of corticosteroids may be important in selected post‐transplant patients with recurrent or antimicrobial‐refractory epiglottitis.

## Author Contributions

R.N. provided clinical care, collected data and wrote the paper. K.O. provided clinical care and edited the manuscript. H.K., M.W., H.A., E.O., H.K., T.M., A.K., and S.H. provided clinical care. R.K. provided clinical care and supervised the figures. N.F. and H.H. provided clinical care, edited the manuscript, and supervised the study. All authors reviewed and approved the final manuscript of this report.

## Funding

The authors have nothing to report.

## Ethics Statement

This study was performed in accordance with the ethical standards laid down in the 1964 Declaration of Helsinki.

## Consent

Written informed consent was obtained from the patient for publication.

## Conflicts of Interest

H.H. has received honoraria from Sanofi, Chugai, Novartis, Janssen, AbbVie, and SymBio, as well as research funding from Novartis and educational grants from Eisai, Chugai, Kyowa Kirin, and Sumitomo. N.F. has received honoraria from Chugai, Bristol Myers Squibb, and SymBio, as well as research funding from IQVIA, Incyte, Chordia, AbbVie, Chugai, Kyowa Kirin, Loxo, Takeda, and Genmab. K.O. has received research funding from AbbVie, Celgene, Otsuka, and Pfizer. S.H. has received research funding from Janssen Pharmaceutical.

## Data Availability

The data for this report is available upon reasonable request.

## References

[1] H. Katori and M. Tsukuda , “Acute Epiglottitis: Analysis of Factors Associated With Airway Intervention,” Journal of Laryngology & Otology 119, no. 12 (2005): 967–972, 10.1258/002221505775010823. 16354360

[2] J. C. Murray , J. K. Chiu , S. R. Dorfman , and A. K. Ogden , “Epiglottitis Following Preparation for Allogeneic Bone Marrow Transplantation,” Bone Marrow Transplantation 15, no. 6 (1995): 997–998. 7581105

[3] J. Markowski , G. Helbig , and A. Widziszowska , “Fungal Colonization of the respiratory Tract in Allogeneic and Autologous Hematopoietic Stem Cell Transplant Recipients: A Study of 573 Transplanted Patients,” Medical Science Monitor 21 (2015): 1173–1180. 10.12659/MSM.893267 PMC4423175 25907308

[4] T. El‐Natout , M. A. Natout , and A. L. Hamdan , “Post‐Transplant Lymphoproliferative Disease (PTLD) of the Larynx in an Adult with Kidney Transplant; A Case Report,” Journal of Voice 35, no. 5 (2021): 810.e7–.e8, 10.1016/j.jvoice.2020.01.014. 32151514

[5] J. Surma , T. A. Schneider , J. Cress , and E. J. Krowiak , “Tonsillar Post‐Transplant Lymphoproliferative Disorder Presenting with Hemoptysis after Heart Transplantation: A Case Report and Review of the Literature,” Cureus 18, no. 4 (2026): e107419. 10.7759/cureus.107419 PMC13192383 42181437

[6] J. O'Brien , D. R. Muccio , and J. W. Schrock , “Association of Early Steroid and Antibiotic Therapy With Airway Outcomes in Adult Epiglottitis: A 10‐Year Multicenter Retrospective Cohort Study,” Academic Emergency Medicine 33, no. 4 (2026): e70285, 10.1111/acem.70285. PMC13103921 42023439

[7] E. Guardiani , M. Bliss , and E. Harley , “Supraglottitis in the Era Following Widespread Immunization Against *Haemophilus influenzae* Type B: Evolving Principles in Diagnosis and Management,” Laryngoscope 120, no. 11 (2010): 2183–2188, 10.1002/lary.21083. 20925091

[8] L. A. Guldfred , D. Lyhne , and B. C. Becker , “Acute Epiglottitis: Epidemiology, Clinical Presentation, Management and Outcome,” Journal of Laryngology & Otology 122, no. 8 (2008): 818–823, 10.1017/S0022215107000473. 17892608

[9] D. Ion , K. Stevenson , and S.‐B. Woo , “Characterization of Oral Involvement in Acute Graft‐Versus‐Host Disease,” Biology of Blood and Marrow Transplantation 20, no. 11 (2014): 1717–1721, 10.1016/j.bbmt.2014.06.031. 24979731

[10] N. Nakamura , T. Jo , and Y. Arai , “Severe Cases of Local Cytokine Release Syndrome (CRS); Craniocervical Edema Soon After Chimeric Antigen T‐Cell (CAR‐T) Therapy,” Oxford Medical Case Reports 2025, no. 1 (2025): omae164, 10.1093/omcr/omae164. PMC11748437 39839700

[11] C. Luan , J. Zhou , and H. Wang , “Report: Local Cytokine Release Syndrome in an Acute Lymphoblastic Leukemia Patient after Treatment with Chimeric Antigen Receptor T‐Cell Therapy: A Possible Model,” Literature Review and Perspective Front Immunol 12 (2021): 707191. 10.3389/fimmu.2021.707191 PMC8326907 34349766

[12] H. Shima , T. Kurosawa , and H. Oikawa , “Cervical Edema Extending to the Larynx as Local Cytokine Release Syndrome Following Chimeric Antigen Receptor T‐Cell Therapy in a Boy With Refractory Acute Lymphoblastic Leukemia,” Case Reports in Oncology 15, no. 1 (2022): 257–262, 10.1159/000522669. PMC8958609 35431861

[13] M. Alvarez de Linera Alperi , S. Ferran de la Cierva , M. Palacios Berraquero , D. Terrasa Czapiewska , A. Alfonso , and S. Fernandez Gonzalez , “Severe Laryngeal Edema After CAR‐T Cell Treatment in a Patient With Multiple Myeloma: A Case Report,” Clinical Case Reports 11, no. 6 (2023): e7281. 10.1002/ccr3.7281 PMC10242084 37287622

[14] Y. Aono , S. Imokawa , T. Uto , J. Sato , F. Tanioka , and T. Suda , “Granulomatosis With Polyangiitis Involving the Epiglottis,” Respirology Case Reports 5, no. 3 (2017): e00226, 10.1002/rcr2.226. PMC5331755 28261483

